# “Ways and channels for voice regarding perceptions of maternal health care services within the communities of the Makamba and Kayanza provinces in the Republic of Burundi: an exploratory study”

**DOI:** 10.1186/s12913-017-2822-y

**Published:** 2018-01-29

**Authors:** Prosper Niyongabo, Renate Douwes, Marjolein Dieleman, Frédéric Irambona, Jimmy Mategeko, Georges Nsengiyumva, Tjard De Cock Buning

**Affiliations:** 1 Institut National de Santé Publique, Avenue de l’Hôpital 3, Bujumbura, Burundi; 2 Fonteinlaan5, 2012 JG, Haarlem, The Netherlands; 3 Royal Tropical Institute, Mauritskade 63, 1092 AD Amsterdam, the Netherlands; 4 CORDAID-BURUNDI, Avenue du Marché 9, Bujumbura, Burundi; 5 CARE-BURUNDI, Avenue Mwezi Gisabo 30, Bujumbura, Burundi; 6 Institut National de Santé Publique, Avenue de l’Hôpital 3, Bujumbura, Burundi; 7 Vrije University of Amsterdam, De Boelelaan 1085, 1081 HV Amsterdam, The Netherlands

**Keywords:** Maternal health, Social accountability, Voice, Community mobilization, Community empowerment, Participatory approaches

## Abstract

**Background:**

Increased availability of maternal health services alone does not lead to better outcomes for maternal health.The services need to be utilized first.One way to increase service utilization is to plan responsive health care services by taking into account the community’s views or expressed needs.

Burundi has a high maternal mortality ratio, and despite improvements in health infrastructure, skilled staff and the abolition of user fees for pregnant women,utilization of maternal health services remains low. Possible reasons for this include a lack of responsive healthcare services.

An exploratory study was conducted in 2013 in two provinces of Burundi (Makamba and Kayanza), with the aim to collect the experiences of women and men with the maternal health services,their views regarding those services, channels used to express these experiences, and the providers’ reaction.

**Methods:**

Semi-structured interviews were used to collect data from men and women and key informants, including community health workers, health committee members, health providers, local authorities, religious leaders and managers of non-governmental organizations. Data analysis was facilitated by MAXQDA 11 software.

**Results:**

Negative experiences with maternal health services were reported and included poor staff behavior towards women and a lack of medicine. Health committees and suggestion boxes were introduced by the government to channel the community’s views. However, they are not used by the community members, who prefer to use community health workers as intermediaries. Fear of expressing oneself linked to the post-war context of Burundi, social and gender norms, and religious norms limit the expression of community members’ views, especially those of women. The limited appreciation of community health workers by the providers further hampers communication and acceptance of the community’s views by health providers.

**Conclusion:**

In Burundi, the community voice to express views on maternal health services is encountering obstacles and needs to be strengthened,especially the women’s voice. Community mobilization in the form of a mass immunization campaign day organized by women fora, and community empowerment using participatory approaches could contribute towards community voice strengthening.

**Electronic supplementary material:**

The online version of this article (10.1186/s12913-017-2822-y) contains supplementary material, which is available to authorized users.

## Background

Reducing maternal mortality and morbidity remains a challenge worldwide. In 2013, 289,000 women died from pregnancy-related causes, and maternal death was identified as the second “biggest killer” of women of reproductive age for that year [1]. The majority of those deaths occurred in developing countries, especially in sub-Saharan Africa, and could have been prevented by improving access to quality care. In line with this, skilled birth attendance was recognized as a crucial intervention to reduce maternal mortality and morbidity [2, 3]. However, skilled birth attendance cannot be achieved if maternal services are not sufficiently utilized. If health services were more responsive to the citizens’ demands and made changes on the basis of ideas or concerns raised by or with community members [4], their utilization would probably improve [5]. A key way to ensure better utilization of services is to strengthen the community voice and take into account community concerns and views in health planning through social accountability [6]. Social accountability can be defined as “an approach towards building accountability that relies on civic engagement in which it is ordinary citizens and civil society organizations who participate directly or indirectly in exacting accountability” [7]. In a number of countries such as Uganda [8], India [9] and South Africa [10], experience has shown the potential of social accountability to improve service delivery and policies. For social accountability mechanisms to be effective and lead to concrete results such as responsive services, the citizens need to have a voice, which refers to the capacity for people to express their complaints and views collectively [6]. Burundi has a maternal mortality ratio of 500 per 100,000 live births [11].This high ratio is one of the consequences of the disruption of the health system due to 13 years of civil war that ended with a peace agreement in 2005. Burundi is thus a post-conflict country, and since 2006, the government has invested in health system improvement and accessibility. In this line, the government introduced free health care for pregnant women and performance-based financing of health facilities (PBF) to enhance health service provision. Despite these changes, the utilization of maternal health services, including institutional deliveries, remains low. According to the most recent Demographic and Health Survey carried out in 2010 [11], 40% of deliveries occur at home, only 21%of pregnant women attend their first antenatal care (ANC) visit early (8–12 weeks of pregnancy) as recommended by the World Health Organization (WHO) [12],13% of women aged between 15 and 49 years and 22% of women in union use family planning methods, while 31% of women in union have unmet needs for family planning .

The quality of maternal health services, such as antenatal care, family planning, delivery and postnatal care (PNC) services is perceived by both women and men as poor.

The main reason is that cultural views on maternal and reproductive health are not taken into account [13]. Therefore, women and their family opt to seek care first within their community and not at a health facility [14]. Civil society organizations and health committees are structures that can be used to discuss the health services user’s views on health issues [15, 16], but it is not clear if and how women and men currently express their views.

This article presents the findings of a study on community perceptions of the quality of maternal health services and on the ways used to express these perceptions and related concerns in order to discuss opportunities for community members to use voice for social accountability regarding maternal health care in Burundi. The study findings presented in this article reflect the situation in Burundi from 2006 to the end of 2014, before the 2015 political crisis around the presidential elections.

## Methods

### Study design

This study was a qualitative, exploratory study using semi-structured interviews that aimed to answer the research question: “What are the community’s perceptions on maternal health services, and what are the ways used by the community members to express them?” Issues relating to the communities’ experiences with maternal health care, channels used to express concerns and views, factors influencing their expression, and the provider’s responsiveness were examined.

### Selection of study sites and respondents

The study was carried out in two provinces of Burundi: Makamba and Kayanza. The choice for these provinces was guided by the presence of key research partners: the non-governmental organizations (NGO) CORDAID (in Makamba) and CARE International (in Kayanza).

In Makamba, CORDAID is implementing a Performance-Based Financing (PBF) scheme at the health service and community level, which includes efforts to improve voice as health committees participate in preparing the development plan of health centers together with health providers, while Community-Based Organizations carry out a quarterly survey among people who have used the health services so as to rate service performance from a community perspective.

**Fig. 1:**
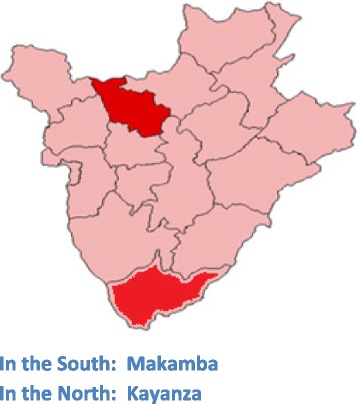
The location of Makamba and Kayanza provinces. In the South: Makamba. In the North: Kayanza

In Kayanza, CARE Burundi uses a community score card to ask people to rate the health services and get the communities and health providers to discuss the results together, with the aim to improve governance within health sector. Figure 1 shows the location of Makamba and Kayanza provinces in BURUNDI.

Within each province, two health centers (HC) were chosen using two criteria: operating at the average provincial outpatient utilization rate of 2012 (MoH, 2013 unpublished data) and location in different environments.

In Makamba, a health center located on the plains near Lake Tanganyika and one located in the mountains were selected. The assumption behind this choice was that people living on the shores of Lake Tanganyika have regular contact with outsiders because of trading with people from the capital Bujumbura and from Tanzania and thus are used to expressing their views more openly than the more isolated people living in the mountains.

In Kayanza, a health center on the outskirts of the town of Kayanza was selected and another one in a remote rural plain. Kayanza is a provincial trading center interacting with Rwanda and the capital Bujumbura and hosting many governmental services. People in the rural plain are isolated and lack these exchanges. As in Makamba, we expected the people in the trading center to express themselves more freely.

The study population comprised men and women of reproductive age. Key informants were selected among health care providers, religious leaders, health committee members (HCMs), community health workers (CHWs), NGO employees, and authorities at the local and provincial level.

Initially, we intended to use purposive sampling for the community members. However, when we approached women, we noticed that few of them would talk openly about social accountability in relation to maternal health care. We therefore decided to use convenience sampling. Men and women of reproductive age were recruited in the catchment area of the HC in public spaces. Participants were approached on the streets and at small rural markets, and those who were willing and had time were interviewed. Due to their reluctance to speak out, we interviewed fewer women than planned.

The inclusion of CHWs, HCMs, and health care providers was facilitated by the in-charge of the health facility. At least one nurse working in the maternal health ward within each of the four health centers included in the study was interviewed.

From NGOs, the programme managers or programme executive officers were selected. From religious organizations, the local community representatives who were available and present during the time of data collection were interviewed. Authorities were also interviewed in their office by appointment.

In total, 138 respondents were interviewed:27 men, 19 women, 8 community leaders, 36 community health workers (CHWs), 12 health committee members (HCMs), 5 local representatives, 21 health providers, 5 NGO staff members, 5 religious leaders. The majority of men, women, CHWs, HCMs are people from rural areas, living of agricultural activities and having a low educational level. Few women were in a leading position within the community. The majority of health providers were nurses. Respondents were Catholics, Pentecostals, Methodists and Muslims. Religious leaders were also from those religious communities. Table 1 provides a large overview of the respondents’ characteristics.

**Table 1:** The respondent’s characteristics

Characteristics and total number	Women:19	Men: 27	CHW: 36	HCM: 12	Religious Leaders: 5	Decision-makers: 5	Community leaders (=chief of colline): 8	NGO staff: 5	Health care providers: 21
Age									
≤20	2	0	0	0	0	0	0	–	0
20–35	13	10	5	4	0	1	1	1	11
35–50	4	6	28	6	4	4	1	3	6
>50	0	11	2	2	1	0	6	1	0
Not reported	–	0	1	0	–	–	–		4
Matrimonial status									
Married	16	26	34	12	4	5	7	4	15
Divorced	0	0	0	0	0	0	0	0	0
Single	3	0	0	0	1	0	0	1	6
Widowed	0	1	2	0	0	0	1	0	0
Educational level									
Primary school (not completed)	8	14	28	5	1	0	8	0	0
Did not complete secondary school	6	2	6	3	0	0	0	0	0
Completed secondary school	0	0	0	2	1	0	0	2	18
University	0	0	0	1	2	5	0	3	3
Completed informal education	4	8	1	0	1	0	0	0	0
No education	1	2	1	1	0	0	0	0	0
Years in the current task and profession for health care providers			≤5:8 5–10:22 >10:3 Missing:3	≤5:6 5–10:4 > 10:2			≤5:3 5–10:4 >10:0 Not reported:1		≤5:11 5–10:6 >10:2 Not reported:2
Not reported		1	–	–					
Profession									
Farmer	19	27	36	9	–		8	0	0
Civil servant	0	0	0	3	–	5	0	0	21
Programme manager								5	0
Not reported					5				–
Religion									
Catholic	12	21	24	8	1	–	8	–	–
Pentecostal	5	1	10	1	2	–	0	–	–
Living church	1	2	0		1	–	0	–	–
Anglican church	1	1	2		0	–	0	–	–
Muslim	0	1	0	1	1	–	0	–	–
Jehovah’s witnesses	0	1	0		0	–	0		–
Not reported				2		5		5	21
Gender									
Female	19	0	13	4	0	1	2	0	12
Male	0	27	23	8	5	4	6	5	9
Number of children									
0	0	0	–	–	–	0	0	–	–
≤3	11	9	–	–	–	3	0	–	–
>3	8	15	–	–	–	2	6	–	–
Not reported		3	36	12	5	0	2	5	21
Place of residence									
Makamba	11	12	23	7	2	2	4	4	11
Kayanza	8	15	13	5	3	3	4	1	10

### Conceptual framework

#### Social accountability

To show how voice is related to social accountability and how social accountability could influence responsiveness, the conceptual framework of social accountability proposed by Lodeinstein et al. was used [17]. It distinguishes 3 elements:

1. Citizen engagement, which includes participation and voice or expressing their expectations and concerns collectively, but without formal ways of enforcing that they are taken into account.
2. Citizen oversight, which includes involving citizens in monitoring and evaluating health services and the performance of health service providers, sanctioning when poor performance occurs and rewarding when performance is perceived as good.
3. Both are required to produce responsive health services, contributing to improved health and social status and rights.

**Fig. 2:**
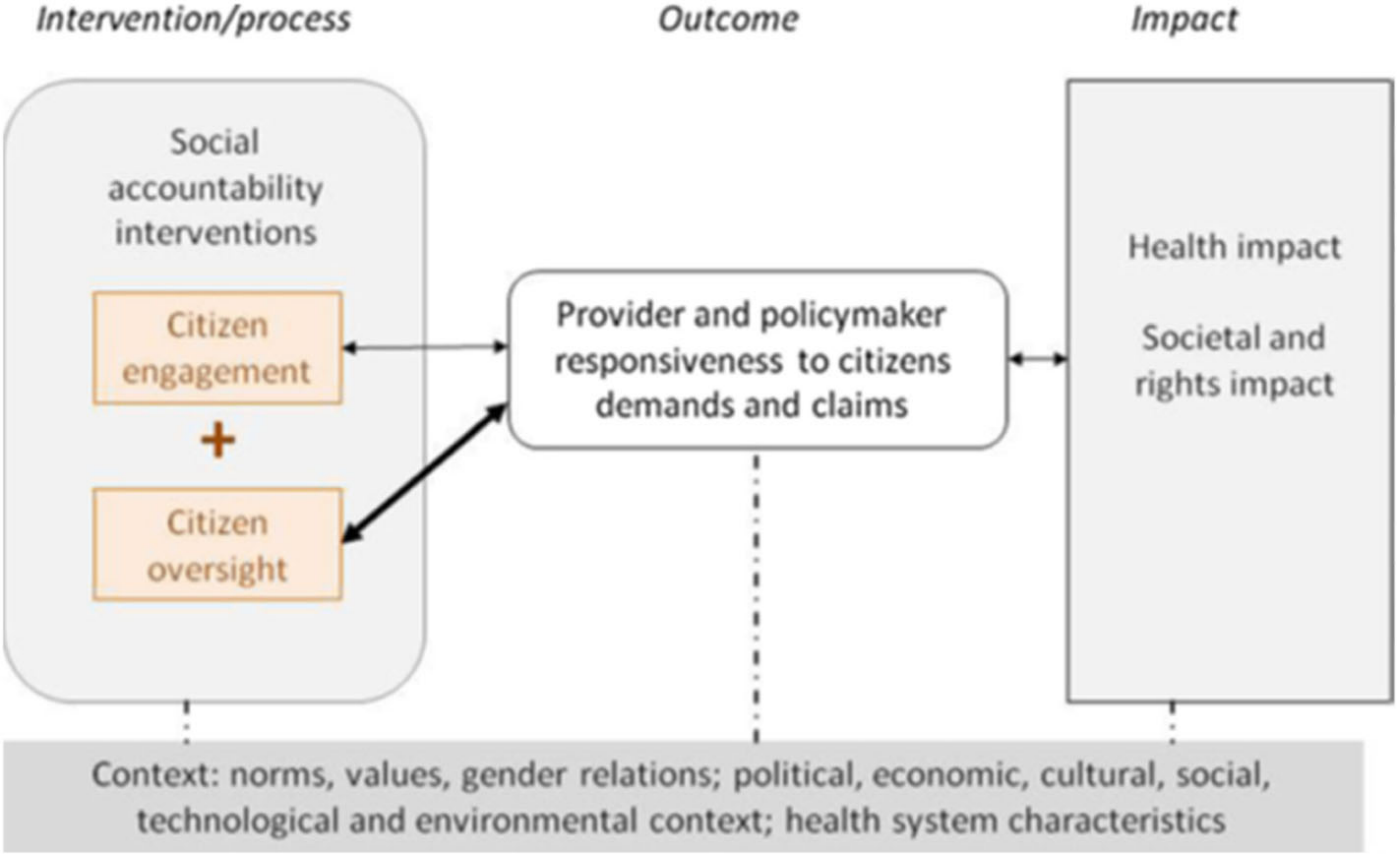
Conceptual framework for social accountability

In this study, we explore the current use of voice as it constitutes a precondition to implement social accountability, which in turn will lead to greater responsiveness of the maternal health services. This is visualized in Fig. 2.

### Data collection

Data were collected through semi-structured interviews. An interview guide was prepared and adapted to each group of respondents (Additional file 1). The interview guide was pre-tested in Makamba, in a health facility and a community outside the study sites. The interview guides were developed in French and translated into Kirundi by the researchers. Data collection was performed by a team of 3 researchers trained in qualitative research. This team consisted of a principal investigator (PI), who is a public health researcher at the National Institute of Public Health in Burundi and a PhD student at VU University of Amsterdam, a Dutch female researcher from VU University of Amsterdam, and a Burundian female research assistant acting as both interviewer and translator. Interviews were held in the local language (Kirundi) except for NGO personnel, provincial-level authorities, and the health center in-charge, who were interviewed in French. Interviews were held until data saturation was reached, that is, no new insights were gained through the interviews. Data collection was carried out from 2 to 30 October 2013.

### Data analysis

Data analysis started with verbatim transcription of the audio-recorded, anonymized interviews and translation from Kirundi into French by a team of local translators. Translations were checked for quality assurance by the PI. The data coding was performed using MAXQDA 11 software. Researchers worked in a team to identify themes related to the research objectives in the transcripts. Based on commonly described themes and subthemes, the researchers individually coded the transcripts. When a new theme or subtheme emerged, the team discussed it and agreed on its inclusion. The main themes identified were related to the main research question: views on the quality of maternal health care services, channels used by women and men to express their views and concerns, obstacles to voicing views, and reactions of providers to views expressed by the community. The data were triangulated by asking the same questions of different groups of respondents and by reviewing documents such as national policies and reports from the Ministry of Health.

## Results

Although the study was carried out in two different provinces of Burundi, the results are presented together as no major differences were found between statements made by men and women and key informants from Kayanza and Makamba. The results are presented according to the four main themes addressed in the interviews: views on the quality of maternal health services, obstacles to voicing them, channels to express views, and perceived providers’ responsiveness.

### Views on the quality of maternal health care services

This section presents the perceptions of different groups on the quality of the maternal health services. Table 2 gives an overview of the main issues raised in the two provinces.

**Table 2:** Overview of main perceptions per group of respondents on maternal health care quality

Group	Perceptions
Women	Nurses late or absent; delays in the facility; poor attitude towards patient, shouted at them or ignored them; payment required when seeking care for side-effects due to family planning method
Men	lack of medicines and the general high cost of medical services; parallel sales of drugs
CHW	Case of women who were not received at health facility
HCM	Privacy, rudeness, physical abuse, long waiting time before being seen
Community leaders	Poor staff attitude
Health providers	Long waiting time for patients
Authorities	Staff attitude towards women is bad.

In general, negative experiences with the quality of maternal health care services were more frequently expressed than positive ones. In both provinces, most of the respondents started sharing a positive experience but later on added a negative experience. Frequently expressed positive experiences by women included the feeling of having been well taken care of at the facility during delivery. Women mostly expressed negative experiences in which nurses were late, absent, delayed, had a poor attitude towards women, shouted at them or ignored them, as illustrated by the following quote: “*They receive you, but very often… when we call the nurse he/she takes a long time to arrive, and you can even find a woman delivering on the floor”* (woman, Makamba). Other negative experiences included poor quality of antenatal consultations with little information about the fetus and paying for the treatment of side-effects linked to the use of family planning methods that are provided for free. Men also expressed concerns about health care provision in their health centers. This fragment illustrates their general feeling: *‘they [the providers, ed.] don’t work well, they come in too late, they leave early, they are absent, they insult the patients, for example when a woman is about to deliver she is not treated well and suffers. This all raises a feeling of dissatisfaction among the population*’ (man, Kayanza). Men emphasized the lack of medicine and the general high cost of medical services. They believe that medical personnel ask high prices and keep a share for themselves, *‘We think that the medicines are sold. For example, the liquid medicines (…) when you go to the pharmacy you’ll find a lot of different categories. We wonder how and why these medicines are not available at the health center*’ (man, Makamba).

The poor attitude of staff was the complaint most frequently reported by community leaders, and they mentioned cases where women were refused admittance at health facilities. Provincial medical and political authorities acknowledge that the attitude of medical staff towards patients and their behavior need to improve. They declared that they regularly hear people complaining about the way they are received by the medical staff and that there are even some people who are shouted at or insulted.

Some HCMs pointed out the poor attitude of health providers as well, for instance rude nurses and cases of women who were abused. *‘There were some celibate nurses who showed a regrettable behavior. Pregnant women were afraid to have them help during the delivery because these nurses revealed everything they had seen to the whole community’*(HCM, Makamba). CHWs stressed the long waiting times at health facilities and reported cases of women who were not received at health facilities. “*A pregnant woman came here to the HC for delivery. The nurse refused to receive her. Then she went to seek accommodation in the vicinity of the health center and delivered there helped by the family who received her…*” (CHW, Kayanza). According to the CHWs, health providers do not easily accept patients who come to the health facility in the afternoon or at the end of the day. They want them to come in the morning. Health providers admitted that nurses do not always work in accordance with standards. *‘It happens that a nurse arrives too late during the night. This is not the rule; the rule is that the nurse waits for the patients and not the other way around’* (health care provider, Makamba). Different causes for this were mentioned such as a high workload, health provider’s negligence, or sickness.

### Obstacles and problems with voicing views with regard to poor quality of care

Few men or women reported having addressed their concerns directly to the health providers. A woman from Makamba was among those who dared to do so. She said, “…*the nurse prescribed drugs for me and my newborn without any testing. I directly protested and asked for biological tests, but the nurse refused and I went home*…” The reasons given by those who did not dare to confront the health provider directly when they experienced poor treatment at a health facility were related to hierarchical social relations, gender, political factors, religion and the lack of feedback about voiced complaints.

#### Hierarchical relations

Fear of the higher social status and social consideration of the health providers was reported to have deterred women and men from discussing their views about received care directly with the health providers. A man from Kayanza explained, ‘*For us, the simple citizens, it is difficult, we are afraid to confront the nurses who have a higher social status than us’* (man, Kayanza).

#### Gender

Women were reluctant to speak out, and it took time to interview them. Those who consented to an interview reported a fear of expressing their views publicly. A woman from Makamba shared her reasons not to speak out publicly. *“I am afraid to speak out publicly because I think I will appear ridiculous”(*woman, Makamba). Other women declared that they do not feel entitled to attend meetings and voice their concerns or confront health providers’ views because of their social status as illegal spouses, having children outside of marriage or being poor. An interview with an unmarried woman with children in Kayanza illustrates this. ‘*I thought I did not have the right to attend because I was pregnant without being married.’* Another woman from Kayanza declared, *“I cannot complain about care received or confront health provider’s views because I am poor.”*

#### Political factors

Participants reported political challenges to the free expression of their views. A religious leader in Makamba said, *“We are not a democracy, and free expression has not been in our country for many years. People cannot speak for themselves.*” Political influence was also mentioned by some of the respondents within the health sector. For instance, a man who lost the election for HCM in Makamba declared, *“During the last health committee elections, there was a very big political influence, and we could see that the majority of elected HCM consisted of members of our main ruling party.*”

#### Religion

The study revealed that religion was exerting an influence on citizens’ expression of their views. Some respondents, especially Roman Catholics, Pentecostals and Methodists, reported that they were afraid to take a position or act in a way that contradicts religious views. An example to illustrate this situation is family planning; some couples in need of birth control fear to use contraceptives because their religion forbids this.

A woman from Makamba gave the following reason. *“Our pastor told us that God said that every woman has to give birth to as many children as possible.”* Some women and men stated that there are some church members who report women who go for family planning to the pastor. Even health providers assumed they were aware of it as one of them declared, *“There are women here in the hospital who report to their pastor the names of women who come to seek family planning methods”* (health care provider, Makamba). When asked if he thinks that some of his church members go for family planning, a religious leader answered, *“In our Pentecostal church, we are a small community. No one can cheat and go for family planning as it will soon be visible*” (religious leader, Makamba).

#### The lack of feedback from health providers to the community

The lack of feedback to the community was discovered in this study. Community members mentioned that they do not receive feedback after expressing views, expectations, or concerns. In Makamba, for instance, citizens complained about the lack of feedback after complaining about the misbehavior of a nurse. This fragment of an interview with a man in Makamba illustrates the problem.

*“There was a case of a nurse who attempted to rape a woman at the health center. This case was reported to the health center in-charge.* Researcher: *How was he punished?* The man: *We do not know, because we did not see him again.* Researcher: *Do you think he was punished?* The man: *I do not think so.”* The wish to get feedback for expressed complaints was also pointed out by a woman in Makamba. She said, “*We would like to have opportunities to express our wishes but also get results of expressed complaints instead of dying with anxiety*.”

### Channels used by women and men to express their views

When women and men decide to speak up, they report that the following channels (or structures) could be used.

#### Use of intermediaries

##### Health committee members (HCMs)

In Burundi, a policy on community participation has existed since 2011 [16], which requires that each health center must establish a health committee tasked with expressing community health needs, helping the health center in establishing health priorities, dealing with health promotion, financially co-managing the health center, establishing an annual action plan together with the medical staff, identifying indigents within the community, and speaking on their behalf at the health facility. HCMs are people from the community, elected by the population of a catchment area of a health center. Elections are organized at *colline*^1^ level and are supervised by the health center in-charge and the chief of the colline. Conditions for eligibility include being a permanent resident of the health center catchment area, being literate, being known as an honest person, not being a CHW, and demonstrating respectful behavior within the community. HCMs are elected for a renewable term of 5 years [16].

However, despite this national policy, many citizens (especially women) do not seem to know their HCMs and do not use them as messengers to express their complaints, as illustrated by the following quote. *“I think there are authorities. I have lived here for three years but have never seen them”* (woman, Makamba). In Kayanza, men declared that they feel they are not in a position to talk to a HCM as illustrated by this answer, *‘I heard about health committee members, but I do not think I can talk to them since they are educated people*’ (man, Kayanza).

##### Community health workers (CHWs)

In its policy for community participation, the Ministry of Public Health outlined the role of CHWs [16]. CHWs operate at the community level with a particular emphasis on health promotion activities, informing women about institutional delivery and directing them there. Each *subcolline*^2^ must have one CHW. Three or five subcollines make up a colline. At this level, equal numbers of female and male CHWs have to be present. CHWs are people from the community elected by community members under the supervision of the health center in-charge and the chief of the colline. Criteria for eligibility are age between 20 and 50 years, completed primary school, residing within the community, and being an honest person. Candidates should not be a HCM, a civil servant, or an employee of a company. They are elected for an unlimited period of time [16].

Men and women mentioned CHWs as a channel that can be used to report their grievances about (maternal) health provision. A woman said, *“I always submit my complaints about health care provision at the health center to community health workers because they are the ones who can channel them towards upper levels*” (woman, Makamba). The extent of their influence on health providers is unknown, however. A man stated, ‘*I don’t know if they are capable of changing something at the HC’* (man, Kayanza). Most health providers confirmed the role of CHWs as messengers for conveying concerns. However, the majority of CHWs themselves do not consider their main role as being messengers for the population. They consider themselves as providers, as one of them stated, “*I am a CHW. My role is to accompany and direct women to the health center and to advise them about good practices of pregnant women”*(CHW, Makamba).

The work of a CHW seems not always to be accepted by health providers because of their low level of education, as one female CHW reported, “*Health providers welcome us in a friendly way*. *The problem comes when the community health workers cannot read and write. Providers ignore them and do not consider them”* (CHW, Kayanza).

##### Chiefs of collines

Women and men were of the opinion that chiefs of “collines”are not the first people to turn to with health concerns. They are approached in cases of social conflicts. During community meetings, health concerns are expressed and then reported to the health center in-charge by the chief of colline. However, feedback is not usually given to the community leader, who could inform the community.

One chief of colline declared, ‘*When we tell the Health Center in-charge, he says we have to point out the nurse who didn’t perform well. We give the name of this person, but I don’t know if the Health Center in-charge discusses this issue with the nurse’* (chief of colline,Kayanza).

#### Other

##### Gossip

Because of their fear of speaking up, women gossip with neighbors and friends at the health facility to express their feelings. When asked about ways used by women to express their complaints, a woman in Makamba answered, “*Women can gossip about a health provider with friends at the health center, hoping that someone among those who hear the story could tell it to that health provider*” (woman, Makamba).Gossip is an informal and traditional way to convey a message indirectly and anonymously to someone afraid for his/her social status or authority within the society. It is always cautiously done within a group of people with the hope that the message will spread out and reach the person and make him/her change a procedure or a behavior causing concern or dissatisfaction. In Burundi, gossip has been used in the past as a space to contradict the authority’s views and policies and is considered a threat to national security [18].

##### Loud speaking

Some women speak in a loud voice at the health center to get the provider’s attention. This quote illustrates it, ‘Women speak in a loud voice in the Health Center, hoping that the providers will hear them’ (woman, Makamba).

Others discuss loudly in a group, thinking that health providers or another member of the staff can overhear them. This woman from Kayanza explained it clearly, “*The health providers can overhear us when we complain in a group at the health center*”. Speaking loudly within the health center seems cautiously done, however. A woman in Kayanza reported that it was easier for her to speak loudly outside the premises of the health center than inside. She said, “*I am afraid to speak loudly against the provider in the health center. I only do it at the door of the health center while leaving to avoid being seen by the health provider*” (woman, Kayanza). Other women try to share their experience with people they meet on the road on their way back, hoping that someone among them will tell it to the health provider. This woman from Makamba reported, “*When I am not satisfied with the care received, I try to tell it to anyone I meet in the road on my way back home. Perhaps someone can recognize the health provider I am talking about and tell him or her*.”

##### Suggestion box

As part of a national policy in Burundi(Burundian Ministry in Charge of Good Governance, 2010; unpublished document), suggestion boxes are systematically provided in all health facilities throughout the country as a way to give users the opportunity to express their views about health care provision. However, the community members do not use them. A HCM explained, ‘*Even if they know, they are afraid to put in their suggestions because they fear the nurses will see them* (..) *and they want to avoid the nurses being annoyed with them’* (HCM, Makamba). The health center in-charge emphasizes that the box is frequently opened, but according to him, the population does not understand its use. A nurse in Makamba stressed the fact that illiteracy is a hindrance for the use of suggestion boxes. She declared, “*Suggestion boxes could help the population to express views, but the problem is that there are many people who cannot read and write*”(Health care provider, Makamba). Some men questioned the opening of the boxes and what is done with the suggestions. *‘We don’t see any need for submitting our ideas in there because it will be the same person who opens it as the one you’ve talked about”* (man, Kayanza).

### Reactions of providers to views expressed by the community

The majority of health care providers in both provinces had a negative attitude towards views expressed by citizens. Only a minority of nurses take them positively. For instance, one nurse answered that complaints expressed by the population help in improving the health service delivery, as illustrated by this answer:*“If for example, women complain about old mattresses, we usually organize a meeting to decide upon buying new ones*” (health care provider, Makamba). In both provinces, nurses reported performance problems related to the lack of personnel, equipment, transportation, and medicines.

In Kayanza, some nurses felt frustrated: *‘They often complain that we receive them too late, or judge us on not being concerned for the patients, but that doesn’t derive from our unwillingness but from a lack of personnel’ (*health care provider, Kayanza).

In the two provinces, many nurses reacted in a more negative way, for example this nurse from Kayanza who declared, “…*complaints will always be there. As a nurse, I have rules to follow. The client has his/her own way of thinking. Understanding each other is difficult* …” (health care provider, Kayanza). Health center in-charges were also asked about how they deal with views and concerns from citizens directed at one of their colleagues. An in-charge declared that community views are usually discussed during staff meetings in order to find solutions. He added that official laws apply in problematic situations. He gave the following example. *“I received a case of a nurse who was misbehaving towards patients. I asked him to give an official explanation, and afterwards he was sent by the district medical officer to another health center as an official sanction*” (health care provider, Makamba). A medical doctor in charge of a district hospital reported having asked a nurse to provide an official explanation after having been accused of stealing drugs that were to be given to a woman.

## Discussion

The study was carried out years before the current political crisis triggered by controversies around the 2015 presidential elections in Burundi. It explored community members’ views and concerns about maternal health care and the channels used to express them. It showed that expressing views in Burundi is challenging because of fear and mistrust due to the post-war status of Burundi, social norms, political and religious influences, and the gender norms limiting women’s expression. It revealed that opportunities for community voice do exist, such as health committees, suggestion boxes, and the use of intermediaries like CHWs. In this section, we reflect on the challenges to community voice and provide a reminder regarding its importance. The section ends with a consideration of opportunities for community voice on maternal health issues in Burundi and some of their limitations.

### Challenges to voice

Community voice faces several challenges in Burundi. The first one is related to political influence, which is fueled by the fact that Burundi was a country where people feared to contradict authorities. The repression of citizen expression has been used by many governments in Burundi since independence as a way to strengthen their power. Control of media, social structures and spaces where people can express their views collectively is common in Burundi. State control of community organizations and structures where citizens can express their views was also found in other African countries like South Africa, where the government limited civil societies’ activities and forced them to act as governmental agencies [19].In Botswana, the government’s decision to incorporate civil society organizations in the state limited community voice as the incorporated organizations could not oppose governmental views [20].In Zimbabwe and Kenya, the functioning of health committees, which are official boards to voice and discuss community concerns in regard to health care, were influenced by political issues [21, 22].

Lack of trust among community members (also one of the characteristics of post-conflict countries) could also have restricted expression in Burundi, as it happened for instance in post-conflict Bissau Guinea [23]. As a consequence, social cohesion cannot be achieved, and collective activities including voicing concerns for better health care cannot be undertaken [24]. Religion is also recognized as exerting a social influence in conflict-affected and post-conflict countries, which could potentially be used to contribute towards establishing peace [25].

Within the health sector, religion can play an important role in voicing community concerns and speaking out on behalf of voiceless and vulnerable people.

This role was particularly seen in fighting stigma against people living with HIV/AIDS (Human Immunodeficiency Virus/Acquired Immunodeficiency Syndrome) and in raising community awareness about HIV [26]. However, religious influence can also limit the community members’ voice as followers do not dare to oppose a decision taken by their religious leaders [27].In the case of religious intolerance, this influence can limit collective activities like voice as people will tend not to interact with people from another religion [23]. In our study, religious leaders opposed the government option for family planning, and followers were afraid to voice their views on the issue individually or collectively for fear of contradicting their religion’s position.

Besides the factors cited above, gender norms and traditions also appear in this study to limit women’s participation in the community’s collective activities. This situation was also reported for other African post-conflict countries where women had to prioritize domestic activities, which are usually excessive because of orphans created by the war [28, 29].

The quality of relations between health providers and the community also influences the community’s voice for health. Examples exist from other countries in Africa (post-conflict or not). In Kenya, for instance, mistrust about the handling of health facility funds led to difficult relationships and even conflict between HCMs and health providers, hampering the discussion over health issues [30]. Our study showed problematic relationships between health providers and CHWs, who are the preferred messengers of the community. One reason could be that health providers hold a higher social status due to their educational level compared with CHWs, who are often poorly educated. In fact, educated people are socially highly valued in Burundi, and education is recognized as a way to social mobility and a strategy to change one’s social status [31].

The last challenge relates to illiteracy, which is common in rural areas in Burundi, and limits opportunities to access to health information and voice - related concerns. Illiteracy was also recognized in studies conducted in other African countries like Nigeria as an obstacle to engaging in group collective activities [32].

### The importance of community voice and engagement for responsive care

The responsiveness of health services towards community experiences and views on health care provision is regarded as a key to improving the utilization of health services [5]. In fact, as demonstrated by Mubyazi in Tanzania [33] and by Bakeera in Uganda [34], the utilization of health services by a community appears to be influenced by its perceptions of those services.

In this study, the majority of health care providers was not open to community views and seemed not to realize the importance of being responsive to patients’ views. The provider’s role in the Burundian health system appears not to be conceived to include listening to community views and taking them into account in the health care planning.

The lack of responsiveness towards patients’ views was also found in other low-income countries by Berlan and Shiffman and seems to originate from the fact that health providers care more about fulfilling conditions defined by the health system than being accountable towards the service’s users [35].

As a consequence, the health services have become underutilized. Examples from Holeta town in Ethiopia showed that fear of mistreatment by health workers contributed substantially to a decrease in institutional deliveries [36], while in the Niger Delta region in Nigeria, the staff’s attitude triggered a reluctance to utilize maternal health services [37].

### Opportunities to express community views in Burundi and their limitations

As shown in our study, despite the adoption of a free care policy for pregnant women, the community (especially women) has concerns about the maternal health care provision. The free health care policy was seen as a means to decrease women’s health-related problems, but the suddenness of its adoption, the ill-prepared state of the health facilities’ staff and managers, and the rapid increase of attendants did not allow for a reduction in women’s health-related problems [38].

Within the Burundian health care system, structures exist for the community to voice its views and concerns regarding (maternal) health care provision.

The first one is provided by health committees, whose functioning has been defined officially since 2011 [16]. Within the PBF scheme that was adopted in 2006 [39] and which is reported by some authors like Bonfrer and colleagues to have improved the quality of services [40], health committees develop health plans for their health centers together with health providers and hence have an opportunity to discuss community priorities and views with them [41]. Apart from health committees as a voice channel, PBF organizes quarterly surveys targeting people who have been using health services and gives them the opportunity to rate their satisfaction about the care received. These surveys are carried out by community-based organizations (CBOs), which are self-established associations operating within the community [41].

The second structure is the suggestion box, which was also provided as a means to gather community views.

However, the effectiveness of these available opportunities for voice is not known. Firstly, the functioning of health committees has not yet been assessed, and the capability of HCMs to discuss health needs with the providers is not known. In addition, the fear of expressing opinions openly and the mistrust and suspicion within the post- conflict Burundian society might deter women from sharing their experiences with health committee members or hamper discussion of those experiences between health committee members and health providers. Secondly, CBO’s surveys involve exit interviews and target only community members who have utilized the health services. They thus bypass the views of those who did not or do not use the facilities. The lack of professionalism in conducting interviews and the context of fear of expressing one’s opinions might lead sometimes to socially acceptable answers. It is worth noting that information provided by CBOs is always directed to purchasing agencies and rarely shared with health staff [41].

Lastly, our study showed that the suggestion boxes are not used by the community. Illiteracy might be the first reason for that as people in the countryside have a higher rate of illiteracy than the 34.1% national average [42]. We also hypothesize that community members do not believe that providers might act beyond their own interest, as it was shown in Uganda [43].

### A way forward for promoting voice in Burundi

To promote community voice and especially women’s voice in Burundi, the community needs to become increasingly active and take a specific role with regard to community voice promotion. This can be achieved by ensuring community participation in maternal health care -related programmes and activities. In this study, community participation is understood in its broad sense as” the involvement of people in a community in projects to solve their own problems “[44]. Community people’s involvement can present as a process towards community empowerment or as a community mobilization. We take Community empowerment as a” process by which people work together at a local or community level to increase the power (control) they have over events that influence their lives” [45] and Community mobilization as” the way in which people can be encouraged and motivated to participate in programmes’ activities” [44]. There has been an increasing recognition particularly in the health field that in order for interventions to be sustainable it is necessary to have community support [46, 47] . For this reason the interest in community participation has been increasing. The term “community participation” has been used as an umbrella term that includes community empowerment and community mobilization.

In Burundi, community voice can be enhanced using both community mobilization and community empowerment. Community mobilization can use women’s forums which are women organizations newly created by the government to address women’s problems. Although community grouping is usually politically sensitive in Burundi, women’s forums can organize meetings with any kind of actors, in a non-political niche. They can nationally mobilize women by organizing a woman- health day in the form of a mass campaign for immunization day [48].

In addition to community mobilization, community empowerment using Participatory Learning and Action (PLA) is another approach that can contribute to the promotion of community voice. PLA focuses on building a partnership between all groups involved in a program to improve health by including intended beneficiaries in the planning, implementation and evaluation of the intervention [49]. Successful PLA depends on building trust and respect among all partners through a facilitated dialogue and discussions. These approaches were reported to have led to improvements in service delivery in settings where they were used. This was the case in the Oyo state of Nigeria where a participatory approach was used for adolescents in a reproductive health programme to discuss needs and agree on priorities [50]. In Eritrea, Somalia, and Mozambique, the War-Torn- Societies project, which helps societies emerging from war to cope with the challenges of societal and country reconstruction by bringing together local actors, including former adversaries and victims, and international actors, used dialogue within a participatory study and reached consensus on key priorities, and this helped to adapt international aid to local priorities [51]. In Burundi, PLA can take the form of a dialogue between women, health providers and other community actors to discuss maternal health delivery challenges and ways to address them.

However, participatory approaches are reported as being highly context-specific and time-consuming [52]. Based on the latter information and experiences from Nigeria, Somalia, Eritrea and Mozambique [50, 51], the development of such approaches in Burundi should start in local areas, and support from the (local) political and health authorities needs to be sought. When it proves to be successful in the pilot communities, the method can be replicated to other small communities at the local administrative level or to larger communities at a higher administrative level like the province.

## Study limitations

The study has some limitations. Firstly, the absence of differences between the four health centers investigated in the study concerns barriers to community voice and not other aspects of the health system. The authors acknowledge that the four health centers may differ in other aspects. Moreover, although located differently within the province of Makamba and Kayanza, the four health centers are not representative of the whole provincial health system. It is interesting however to note that in our study, barriers to community voice are independent of rural or urban locations, plain or mountain communities, more or less informed communities regarding health services (along trading routes and with access to facilities or not). Nevertheless, this finding cannot be inferred to as no extensive context analysis was carried out beforehand.

Secondly, researchers encountered difficulties related to interviewing women as they were shy and reluctant to express their views on maternal health services. This was mainly due to the facts that researchers were meeting these women for the first time and that women are not used to speak out on the behalf of the community as the Burundian culture and tradition take this as men’s responsibility.

As a consequence, the study led to fewer results than expected for such an exploratory study. However, support for the relevance of their answers came from the contextual actors, husbands and the community key informants.

## Conclusion

Community voice and engagement as preconditions to social accountability are not sufficiently established in Burundi. This study revealed that women and men were limited in expressing their concerns. Formal channels were often not recognized, and opportunities for alternative channels like suggestion boxes were not used. Community voice, especially women’s voice, needs to be strengthened, and at the same time the health providers have to be less defensive and more responsive in order to increase the number of women who will benefit from maternal health services. Recently, the government promoted Women’s Forums as politically neutral niches to focus on the societal problems women encounter. Community mobilization using these women’s forums is proposed to enable community voice with respect to maternal health. Use of participatory approaches to promote women’s voice, like participatory learning and action or interactive learning and action, should also be explored in this context.

## Additional file

Additional file 1: The interview guides. (DOCX 55 kb)

## Abbreviations

AIDS: Acquired Immunodeficiency Syndrome
ANC: Antenatal care
CHW: Community Health Workers
HC: Health Center
HCM: Health Committee Member
HIV: Human Immunodeficiency Virus
HP: Health provider
MoH: Ministry of Health
NGO: Non-Governmental Organization
PBF: Performance-Based Financing
PhD: Doctor of Philosophy
PI: Principal Investigator
PNC: Postnatal care
VU: Vrije University
WHO: World Health Organization

## Footnotes

1 (colline) is an administrative entity within the Burundian administration composed of 3 or 5 subcollines.

2 (subcolline) is a small administrative entity in Burundi hosting around 1000 people. It is used in the health system as a reference for the elections of health committee members and community health workers.
