# Supplementary material for: “Ways and channels for voice regarding perceptions of maternal health care services within the communities of the Makamba and Kayanza provinces in the Republic of Burundi: an exploratory study”
Source: BMC Health Serv Res. 2018 Jan 29;18:46. doi: 10.1186/s12913-017-2822-y (PMC5789700; doi:10.1186/s12913-017-2822-y)
Supplement: Additional file 1: — The interview guides. (DOCX 55 kb) [file 12913_2017_2822_MOESM1_ESM.docx]

**Interview Guides**

1. **Interview guide for the community leaders (chief of colline)**

Good morning,

My name is....................................I am from the VU University of Amsterdam. We are currently caring out a study on the community perception regarding maternal health and ways used to express them as well as community expectations regarding maternal health care. This study will contribute towards improving maternal health care provision in Burundi.

We came here to provide you with opportunity to share your views about the needs regarding maternal health care as members of this community , the way you perceive maternal health care provision in this region, the ways used to express needs in regard with maternal health care as well as people’s expectations for maternal health services. To do so, we are going to ask you questions in regard with the topic.If there are questions or issues for which you do not wish to share your views because of any personal reason, please feel free to skip those questions. Anyway, you can stop this interview at any time you feel you cannot continue.

If you accept this interview, please express your views freely and try to describe the situation as it presents. In fact, your statements will be kept fully confidential and will never be reported to someone else. In this study, each participant will be identified by the mean of a code and the names will never appear. In addition, the information given will only be used by researchers and no one else.

To be able to gather properly your views and thoughts on the topic,we are using a voice recorder.We cannot however record without your consent.The recorder will only be used in case you allow us to do so. Anyway, the records will be erased and transcripts and notes will be kept by the researchers confidentially using codes.

The interview will take one hour. Only volunteers can participate in this study as nothing will be given as a reward to have participated in the study.

Accepting the interview is meant as one way among others to contribute towards improving voice for maternal health care provision in Burundi.

Could we start the recorder and commence the interview?

**Introduction**

Could you tell us about yourself?

-How old are you?

-How far did you go at school?

-Are you married?

-What is your religion?

-How long have you been a community leader?

-What are your duties and responsibities as a community leader?

**Maternal health care**

As a community leader, for which kind of activities in regard with general health care, maternal health and family planningdo you give your contribution?

What do people say about maternal health care provision in this region?

What kind of information do you have in regard with maternal health care provision in this region?

What do you face as challenges in your daily work as a community leader

**Knowledge and experiences with voice mechanisms**

1.What are the ways and social structures used by the community and women to express views, claims and dissatisfaction in regard with maternal health care provision?

2.What kind of information do you have regarding health committees? How do you collaborate with them?

3.What kind of information do you have about community health workers?

4.What are the factors in your opinion than can make people come together and gather as for a meeting?

5.How do you proceed to gather people for a meeting?

6.What do you think are the expectations of the population when they come to participate in a meeting?

7.In your point of view, to which social categories belong people who regularly participate in meetings? What are the categories of people who do not participate? Why?

8.What do you do to convince them to participate in the meetings?

9.What categories or group of women participate regularly in meetings?

10.How and in which circumstances community meetings can contribute towards improving health services in general and particularly maternal health services?

11.What kind of messages get easily acceptance and adherence of the community and for which people easily gather in meeting

**Social aspects in relation with social accountability**

Do you think the Burundian local culture allows women to express freely and openly dissatisfaction during a community meeting? Please explain ?

Do you share the opinion that maternal health care provision in Burundi and especially family planning is in keeping with religious and community believes? Why yes ? Why not?

**2. Interview guide forwomen.**

Good morning,

My name is....................................I am from the VU University of Amsterdam. We are currently caring out a study on the community perception regarding maternal health and ways used to express them as well as community expectations regarding maternal health care. This study will contribute towards improving maternal health care provision in Burundi.

We came here to provide you with opportunity to share your views about your needs as women living in this community, the way you perceive maternal health care provision in this region, the ways used to express needs in regard with maternal health care as well as your expectations for maternal health services. To do so,we are going to ask you questions in regard with the topic. If there are questions or issues for which you do not wish to share your views because of any personal reason, feel free to skip it. Anyway, you can stop this interview at any time you feel you cannot continue.

If you accept this interview, please express your views freely and try to describe the situation as it presents. In fact, your statements will be kept fully confidential and will never be reported to someone else. In this study, each participant will be identified by the mean of a code and the names will never appear. In addition, the information given will only be used by researchers and no one else.

To be able to gather properly your views and thoughts on the topic, we are using a voice recorder. We cannot however record without your consent. The recorder will only be used in case you allow us to do so. Anyway,the records will be erased and transcripts and notes will be kept by the researchers confidentially using codes.

The interview will take one hour. Only volunteers can participate in this study as nothing will be given as a reward to have participated in the study.

Accepting the interview is meant as one way among others to contribute towards improving voice for maternal health care provision in Burundi.

Could we start the recorder and commence the interview?

|  |
| --- |
| **INTRODUCTION** |

Could you tell us about yourself ?

-How old are you?

-What is your religion?

-Are you married?

-How many children do you have?

-How long have you been living here?

-What is your profession or daily activity?

-How far did you go at school?

**Experiences with health services**

-What kind of services are provided for pregnant women at health center?

-Where did you go to get antenatal care and for delivery for your last child?

1. Questions in case of delivery at home.

-Who helped you for delivery?

- What are the reasons for which you decided to deliver at home?

-What people say about the care provided to community and women at health center?

2. Questions in case of delivery at health center

-Could you tell us the way you felt you were treated? Please provide examples as illustration.

-What were your expectations in regard with the care and services provided by nurses and medical doctors?

-Did you get the health care and services you were expecting? If yes, why? If no, why? Please provide examples as illustration.

-What do you think is needed for you to get satisfied with maternal health services?

-What do people say about the services provided by the health center?

-Do you think the health center staff is enough skilled to provide needed care to the community members and women? If yes Why. If No, Why?

- What are the other services provided by the health center in addition to those related the pregnancy?

*-*Have you ever utilized those services? How where you treated? Could you provide examples as illustration?

-What were your expectations from nurses and medical doctors working at the health center?

-Did you get the health care and services you were expecting? Why yes? , why no? Could you give examples as illustration?

-Who else in your opinion can provide the same services in your community?

-What do you think about the opinion stating that traditional healers are better than medical personnel in disease management? Please give reasons.

**Knowledge and experiences in regard with voice.**

-When you have complains regarding the way you were treated by a nurse or a medical doctor to whom do you report the complain? Why?

-Do you think the health center staff will be informed about your complains? Why?

-According to you, whom or which organization deals with your complain?

-With whom do you feel comfortable while expressing yours needs and complains in regard with maternal health care provision? Please explain.

*-*In your opinion, what is a health committee? What is its role?

-Do you think community members can help you in expressing a complaint?

-Do you feel represented by the health committee members?

-Who are community health workers? What do you think is their role?

-Whom do they represent?

-What can they do for you in case you have complaints about the health care provision?

**Attitude towards possible voice channels**

-If you allow me to turn a little bit around, what are the social structures or actors which can receive a complaint in regard with health in general and especially maternal health?

-To which extend do you think it is important to have structures and actors to whom one can report complaints?

-In which circumstances, can they help?

-How can they improve their functioning?

**Women resources and capacity for voice**

-When you have a complain or when you are dissatisfied about the way you were treated by a medical staff member what do you do to express your claim or dissatisfaction

-In your opinion, what do health providers doto know community needs? Could you give examples?What results did it yield?

-What do people in this community when they have complaints about health provision? To whom do they report these complaints?

**Influence of social and cultural factors on voice**

-How is communication between you and health providers?

-Do you find it easy to denounce a bad treatment made by health personnel about yourself while seeking for care? Why? Could you give examples?

-Do you think the population is used to express complaints regarding health services? Ifyes, why? If No, why?

-To which extend women feel comfortable to express claims and dissatisfaction towards health personnel?

-According to you, which religious and cultural believes in contradiction with services provided at health center?

1. **Interview guide for men.**

Good morning,

My name is....................................I am from the VU University of Amsterdam. We are currently caring out a study on the community perception regarding maternal health and ways used to express them as well as community expectations regarding maternal health care. This study will contribute towards improving maternal health care provision in Burundi.

We came here to provide you with opportunity to share your views about your needs as people living in this community,the way you perceive maternal health care provision in this region, the ways used to express needs in regard with maternal health care as well as your expectations for maternal health services. To do so,we are going to ask you questions in regard with the topic. If there are questions or issues for which you do not wish to share your views because of any personal reason,feel free to skip it. Anyway, you can stop this interview at any time you feel you cannot continue.

If you accept this interview, please express your views freely and try to describe the situation as it presents. In fact, your statements will be kept fully confidential and will never be reported to someone else. In this study, each participant will be identified by the mean of a code and the names will never appear. In addition, the information given will only be used by researchers and no one else.

To be able to gather properly your views and thoughts on the topic, we are using a voice recorder. We cannot however record without your consent. The recorder will only be used in case you allow us to do so. Anyway,the records will be erased and transcripts and notes will be kept by the researchers confidentially using codes.

The interview will take one hour. Only volunteers can participate in this study as nothing will be given as a reward to have participated in the study.

Accepting the interview is meant as one way among others to contribute towards improving voice for maternal health care provision in Burundi.

Could we start the recorder and commence the interview?

**Introduction**

Could you tell us about yourself?

-How old are you?

-What is your religion?

- What is your marital status?

- How many children do you have?

-Since when do you live here?

**Knowledge and experiences with health services**

-We are now going to talk about about your experience with health services especially maternal health services as a husband, brother or friend of women

| - What are the services provided for women by the health center? |
| --- |
| -What do people of this community say about maternal health care provision?  -According to what you hear in the community, how do community members especially women rate the performance of services they receive at health center?  -According to you what kind of action can make those services work better?  -What are the other community actors that can provide the same services?  **Knowledge and experience with voice mechanisms**  -When you have claims and complaints about health services what do you do? Why?  -If you have a problem with social services what do you do? Why do you do That?  -Have you already known a situation whereby your sister, wife, or the wife of a friend has been badly treated by medical personnel? Could you provide examples?  -What did you do then ? Why?  -To whom do you direct your complain in such situations ? Why ?  -For which other kind of problems can we use those actors?  -What do you do for other problems not directed to them?  -Who is in charge of solving those problems at health center?  -Who is in charge of those problems at colline level?  -Do you know health committees ?  -What is the role of health committees?In which situation can they help? Why?Do you have examples?  -Do you know health committee members? What is their role? In which situation can they be helpful? Why? Could you provide examples?  **Attitude towards voice mechanisms**  -What kind of social structures or actors to which we can direct a complaint about health especially regarding maternal health?  -How those structures and actors proceed to express those claims you have referred to them?  -In which circumstances those structures and actors can be helpful?  -How can those structures and actors work better?  **Community Resources and capacity to express complains**  -In case you are not happy with the way you were treated by a staff personnel how do you proceed to talk to him/her?  -According to you,what do providers do to know the needs of the community?Could you provide example? What kind of results it can yield?  -When people living in this community experience problems with health providers what do they do? To whom do they direct their dissatisfaction?  **Social and cultural factors influencing voice mechanisms**  -Do women participate in community meetings regarding maternal health? What do you think about this participation?  -Is it easy or difficult for community members to denounce a bad treatment made by health providers? Why? Could you give examples?  -Do you think maternal health services provided in health centers especially family planning are in keeping with religious and community believes ? If yes why? If no why?  **4.Interview guide for deciders (Governor and advisors; administrators of communes)**  Good morning,  My name is....................................I am from the VU university of Amsterdam. We are currently caring out a study on the community perception regarding maternal health and ways used to express them as well as community expectations regarding maternal health care. This study will contribute towards improving maternal health care provision in Burundi.  We came here to provide you with opportunity to share your views about your needs as people living in this community, the way you perceive maternal health care provision in this region, the ways used to express needs in regard with maternal health care as well as your expectations for maternal health services. To do so ,we are going to ask you questions in regard with the topic. If there are questions or issues for which you do not wish to share your views because of any personal reason feel free to skip it. Anyway, you can stop this interview at any time you feel you cannot continue.  If you accept this interview, please express your views freely and try to describe the situation as it presents. In fact, your statements will be kept fully confidential and will never be reported to someone else. In this study, each participant will be identified by the mean of a code and the names will never appear. In addition, the information given will only be used by researchers and no one else.  To be able to gather properly your views and thoughts on the topic,we are using a voice recorder. We cannot however record without your consent. The recorder will only be used in case you allow us to do so. Anyway,the records will be erased and transcripts and notes will be kept by the researchers confidentially using codes.  The interview will take one hour. Only volunteers can participate in this study as nothing will be given as a reward to have participated in the study.  Accepting the interview is meant as one way among others to contribute towards improving voice for maternal health care provision in Burundi.  Could we start the recorder and commence the interview?  **Introduction**  Despite efforts made by the government to improve maternal health services performance and their utilization, figures yield from the Demographic and health survey in 2010 show that maternal health is still poorly established in Burundi:500 deaths per 100.000 live births which means that 5 women per 100.000 thousands live birth die while giving birth. Another figure we have is about health service utilization where we notice that 40% of women are still delivering at home.To address this situation, we are caring out a study to explore ways to strengthen community voice and especially women voice in order to use social accountability mechanisms to improve the performance of maternal health services. We think that this strategy can improve the performance of maternal health services and thus their utilization.  To start the interview we would like to know you more.  Could you tell us about yourself?  -How old are you?  -What is your current function? Since when do you take the function?  -Are you married?  -How many children, do you have?  **Maternal health care**  -As a decider, for which kind of activities in regard with general health care,maternal health and family planning you usually give your contribution?  -What are the important health topics you have already been invited to discuss?  -What do you think since last year could have changed within the maternal health sector and family planning?  -What are your views about the current health policy on maternal health and family planning?  **Experience with voice**  -What are the ways and structures used by the community especially women to express dissatisfaction or a complaint in regard with maternal health services?  -What kind of information do you have regarding health committees?  -What kind of information do you have regarding community health workers?  -What are the ways used by the population to challenge or oppose your decisions in case they are not happy about these decisions ?  -What are the ways ,actors and structures do you use to know women needs in regard with maternal health care?  -What are the ways you use to make a follow up of the use of financial means allocated to maternal health?  **Social and cultural factors influencing community and women voice**  -In your opinion,do you think our tradition and believes can allow women to challenge a decision or express freely and openly a dissatisfaction regarding the provision of maternal health services.?  -Do you find maternal health care provided in our country including family planning in keeping with our religious and cultural believes? Please explain.  **Thanks for the interview.**  **5. Interview guide for health committee members**  Good morning,  My name is....................................I am from the VU university of Amsterdam. We are currently caring out a study on the community perception regarding maternal health and ways used to express them as well as community expectations regarding maternal health care. This study will contribute towards improving maternal health care provision in Burundi.  We came here to provide you with opportunity to share your views about your needs as people living in this community ,the way you perceive maternal health care provision in this region, the ways used to express needs in regard with maternal health care as well as your expectations for maternal health services. To do so ,we are going to ask you questions in regard with the topic. If there are questions or issues for which you do not wish to share your views because of any personal reason feel free to skip it. Anyway, you can stop this interview at any time you feel you cannot continue.  If you accept this interview, please express your views freely and try to describe the situation as it presents. In fact, your statements will be kept fully confidential and will never be reported to someone else. In this study, each participant will be identified by the mean of a code and the names will never appear. In addition, the information given will only be used by researchers and no one else.  To be able to gather properly your views and thoughts on the topic,we are using a voice recorder. We cannot however record without your consent. The recorder will only be used in case you allow us to do so. Anyway ,the records will be erased and transcripts and notes will be kept by the researchers confidentially using codes.  The interview will take one hour. Only volunteers can participate in this study as nothing will be given as a reward to have participated in the study.  Accepting the interview is meant as one way among others to contribute towards improving voice for maternal health care provision in Burundi.  Could we start the recorder and commence the interview?  **Introduction**  Could tell us about yourself?  How old are you?  What is your level of education?  What is your profession?  Are you married?  How many children do you have?  Since when are you member of the health committee?  **Experience and responsibilities**  -What kind of services that are provided by this health center to women?  -What do people say about the services provided to them by the health center?  -As a health committee member what are your actual duties and responsibilities? Whom do you represent?  Who is your boss?  -When did you organize your last meeting?  -What did you discuss during that meeting?  -How can community members get access to your meeting minutes and conclusions ?  -What are the maternal health problems do you face in this community ?  **Experience in regard with voice**  -How do community members get in contact with you in case they want to express their views about health care?  -Could you provide us with examples of complains expressed by the population you have already been dealing with in the past?  -What do you do to respond to those complains?  -What kind of responses do you usually get for complains you expressed on the behalf of the population towards the health center managers?  -How do health providers respond to those claims and views expressed by the community?  -What are options that can be taken to punish the health providers in case of mistreatment of clients?  -Do you know community health workers? According to you, what are their roles? How do you collaborate with them.  **Health committee members ‘ perceptions about the attitude of community members towards them**  According to you, what the community expect from you?  How could you meet their expectations?  **Capacity and resources for voice**  -How do you find the communication between you and the health personnel?  -What are the means do you use to oblige the health center in charge to deal with complains expressed by the population?  -What do you need to work better?  -What would you ask to community as contribution to improve your work?  **Social and cultural factors in relation with voice and social accountability.**  -Is it easy or difficult for community members to denounce a mistreatment done by the health personnel? Why? Could you provide examples?  -Do you think the population can express complaints in regard with health care provision? Why?  -Do you usually receive complains from women? Why Not?  -If yes ,what kind of complaints do you receive?   - According to you, are maternal health services and especially family planning in keeping with religious and community believes in Burundi?Why yes ? Why No?   **6.Interview guide for health care providers (nurses ;medical doctors ;health centers in-charge and hospital in charge)**  Good morning,  My name is....................................I am from the VU university of Amsterdam. We are currently caring out a study on the community perception regarding maternal health and ways used to express them as well as community expectations regarding maternal health care. This study will contribute towards improving maternal health care provision in Burundi.  We came here to provide you with opportunity to share your views about your needs as people living and working within this community,the way you perceive maternal health care provision in this region, the ways used to express needs in regard with maternal health care as well as your expectations for maternal health services. To do so, we are going to ask you questions in regard with the topic. If there are questions or issues for which you do not wish to share your views because of any personal reason feel free to skip it. Anyway, you can stop this interview at any time you feel you cannot continue.  If you accept this interview, please express your views freely and try to describe the situation as it presents. In fact, your statements will be kept fully confidential and will never be reported to someone else. In this study, each participant will be identified by the mean of a code and the names will never appear. In addition, the information given will only be used by researchers and no one else.  To be able to gather properly your views and thoughts on the topic,we are using a voice recorder. We cannot however record without your consent. The recorder will only be used in case you allow us to do so. Anyway,the records will be erased and transcripts and notes will be kept by the researchers confidentially using codes.  The interview will take one hour. Only volunteers can participate in this study as nothing will be given as a reward to have participated in the study.  Accepting the interview is meant as one way among others to contribute towards improving voice for maternal health care provision in Burundi.  Could we start the recorder and commence the interview?  **Introduction**  Could you tell us about yourself?  -How old are you?  -What is your educational level  -Are you married?  -How long have you been working in this health center /hospital  -What is your job? ( nurses; medical doctors)  **Experience with health services**   \| **-**What kind of services do you provide to  pregnant women?mothers and young girls?  -How could you rate the utilization of services  By the population?  -Are you of the opinion stating that women usually use other ways to get care? which ones? Why?  -In your opinion, what are issues in regard with health services here in your facility needing to get improved or changed? \|  \| \| --- \| --- \| \|  \|  \| \|  \|  \|   **Experience with voice and expressing views**  -Are you aware of the community expectations regarding maternal health care services?  -In case you want to know community complains and dissatisfaction in regard with health services functioning, how do you usually proceed?  -Does your facility have ways and mechanisms that can allow you to know the community needs;expectations and wishes regarding you provide to community members?  -In your opinion, what are the ways and actors that could help to channel the patients views to health personnel?  -Do you think the current structures meant to make a bridge between community members and health providers like health committees, associations are properly working? Why?  - In which circumstances can they help women?  -We know that you are working hard and give the best of yourself in providing care but we think that it can happen that someone from the community is not happy with the care you provided to him or her. Have you come across a situation in which someone is not satisfied with the care or services you provided to him or her?  -If such situation has happened to you, how did you know that there was a complaint?  -What do you usually do when you hear that that there is a complaint about the services you have provided yourself?  **Health provider’s attitude towards community voice**  -How would you rate the attention you normally give to opinion and concerns raised by clients?  -To which extend do you think that it is important to have ways by which women can express their opinion about health services and especially maternal health services?  -As a health care provider,do you feel really needed when the health center has to solve conflicts with community members? Why? Why not?  **Ressources , capacities and confidence of medical personnel to express needs and opinion in regard with maternal health care.**  -According to you, what is needed to improve health care provision in this facility?  -How would it contribute towards improving services provision?  - Do you think your facility is enough equipped to provide quality care to women? Why yes? Why No?  -How do you find your workload?  **Social and cultural factors influencing voice**  -Do you think the population is used to express complaints or share views regarding health services provision?  -To which extend do you think women feel comfortable when they express concerns or when they discuss with nurses or medical doctors about health care provision?  - Are you of the opinion stating that there are community or religious believes in contradiction with services you provide in this facility? Which ones?   \|  \|  \| \| --- \| --- \|   **7. Interview guide for Non- governmental organizations’ officers.**  Good morning,  My name is....................................I am from the VU university of Amsterdam.We are currently caring out a study on the community perception regarding maternal health and ways used to express them as well as community expectations regarding maternal health care. This study will contribute towards improving maternal health care provision in Burundi.  We came here to provide you with opportunity to share your views about your needs as people living and working in this community, the way you perceive maternal health care provision in this region, the ways used to express needs in regard with maternal health care as well as your expectations for maternal health services. To do so,we are going to ask you questions in regard with the topic. If there are questions or issues for which you do not wish to share your views because of any personal reason feel free to skip it. Anyway, you can stop this interview at any time you feel you cannot continue.  If you accept this interview, please express your views freely and try to describe the situation as it presents. In fact, your statements will be kept fully confidential and will never be reported to someone else. In this study, each participant will be identified by the mean of a code and the names will never appear. In addition, the information given will only be used by researchers and no one else.  To be able to gather properly your views and thoughts on the topic ,we are using a voice recorder. We cannot however record without your consent. The recorder will only be used in case you allow us to do so. Anyway ,the records will be erased and transcripts and notes will be kept by the researchers confidentially using codes.  The interview will take one hour. Only volunteers can participate in this study as nothing will be given as a reward to have participated in the study.  Accepting the interview is meant as one way among others to contribute towards improving voice for maternal health care provision in Burundi.  Could we start the recorder and commence the interview?   \|  \| **Introduction**  Despite efforts made by the government to improve maternal health services performance and their utilization, figures yield from the Demographic and health survey in 2010 show that maternal health is still poorly established in Burundi:500 deaths per 100.000 live births which means that 5 women per 100.000 thousands live birth die while giving birth. Another figure we have is about health service utilization where we notice that 40% of women are still delivering at home.To address this situation, we are caring out a study to explore ways to strengthen community voice and especially women voice in order to use social accountability mechanisms to improve the performance of maternal health services. We think that this strategy can improve the performance of maternal health services and thus their utilization.  **About the respondents.**  Could you tell us about yourself?  -How old are you?  -What is your educational level  -Are you married?  -How long have you been working in this community?  -What are your responsibilities and duties in your organization?  **Maternal health care** \| \| \| \|  \| \| --- \| --- \| --- \| --- \| --- \| --- \| \|  \| \| **-**What kind of problems the community is facing in the sector of maternal health care and family planning in this region?  -What are the services provided by the health center to women?  -What are the projects or programs relating to maternal health are you currently caring out in this region?  -What kind of challenges are you facing in implementing those programmes or in caring out those projects? \|  \| \|  \| \| **Experience with voice mechanisms and attitude towards social accountability** \|  \| \|  \| \|  \|  \| \|  \| \| - How do you proceed to gather community views especially women views about your programmes? \|  \| \|  \| \| -What are the ways used by the community and especially by women to express complaints or claims in regard with maternal health care provision?   - How does the community proceed to get in contact with you in order to discuss claims or views regarding maternal health care? Could you provide examples of complaints you have been dealing with over the past? - What kind of duties or responsibilities do you hold as international non- governmental organizations in improving maternal health care provision? - What kind of information do you have about health committees? - -How do those health committees function?How do you collaborate with them? - What kind of information do you have regarding community health workers? How do they function? How do you collaborate with them?   **Social and cultural factors influencing voice**   - According to your experience, do women participate in community meeting regarding health care? Why yes? Why Not? - In your opinion, is it easy or difficult for community members to denounce a bad treatment made by a health personnel? Why? Do you have examples? - Do you think that maternal health services provided by non- government within health centers especially family planning are in keeping with religious and community believes? If yes why? Why not? \|  \| \|  \| \|  \|  \| |

|  |
| --- |
| **8. Interview guide for community health workers.**  Good morning,  My name is....................................I am from the VU university of Amsterdam.We are currently carring out a study on the community perception regarding maternal health and ways used to express them as well as community expectations regarding maternal health care. This study will contribute towards improving maternal health care provision in Burundi.  We came here to provide you with opportunity to share your views about your needs as people living in this community ,the way you perceive maternal health care provision in this region, the ways used to express needs in regard with maternal health care as well as your expectations for maternal health services. To do so ,we are going to ask you questions in regard with the topic. If there are questions or issues for which you do not wish to share your views because of any personal reason feel free to skip it. Anyway, you can stop this interview at any time you feel you cannot continue.  If you accept this interview, please express your views freely and try to describe the situation as it presents. In fact, your statements will be kept fully confidential and will never be reported to someone else. In this study, each participant will be identified by the mean of a code and the names will never appear. In addition, the information given will only be used by researchers and no one else.  To be able to gather properly your views and thoughts on the topic ,we are using a voice recorder. We cannot however record without your consent. The recorder will only be used in case you allow us to do so. Anyway ,the records will be erased and transcripts and notes will be kept by the researchers confidentially using codes.  The interview will take one hour. Only volunteers can participate in this study as nothing will be given as a reward to have participated in the study.  Accepting the interview is meant as one way among others to contribute towards improving voice for maternal health care provision in Burundi.  Could we start the recorder and commence the interview?  Could you tell us about yourself?  -How old are you?  -What is your educational level?  -What is your profession?  -Are you married?  - Do you have children? How many ?  -How long have you been community health worker?   \|  \| \| --- \| \| **Work experience and perceived duties**  -As a community health worker, what do you think are your duties and responsibilities?  -Who do you think is your boss?  -In your opinion how your work can help the community?  -What are the difficulties and challenges do you face in your work as community health workers?  -What do you need to improve your work as a community health worker?  **Experience with voice**  -Do you often hear women complaining about maternal health care provision?  -By which way do you get informed about community complains and needs regarding maternal health?  -What do you do then in this case?  -Which ways are used by women to get in contact with you?  -Which ways or opportunities do you use to get in contact with health providers to discuss community complaints?  -Do you know health committees? According to you what is their work and what are their duties?  How do you collaborate with them? \| \| **Perceived community attitude towards community health worker** \| \| -According to you what shows that you are helpful for the community and especially for women?  -Could you provide examples of complaints from community you have already dealt with?  **Resources and capacities for voice**  -What kind of means do you use to oblige the health center in charge to deal with community complains?  -What could do the health providers to allow you to become proactive and efficient in your work?  -What the community members could do to allow you getting more influence and more efficiency in your work?  **Social and cultural factors influencing voice**  In your opinion, what are the social factors within the Burundian context making it difficult to express dissatisfaction or a complain towards the health personnel?  Taking into account our culture and the Burundian society, what are the factors making it difficult for women to express openly a claim or dissatisfaction regarding maternal health?  Do you think that maternal health services especially family planning are in keeping with the religious and the community believes of the community? Please explain. \|  1. **.Interview guide for religious leaders**   Good morning,  My name is....................................I am from the VU university of Amsterdam. We are currently caring out a study on the community perception regarding maternal health and ways used to express them as well as community expectations regarding maternal health care. This study will contribute towards improving maternal health care provision in Burundi.  We came here to provide you with opportunity to share your views about your needs as women living in this community ,the way you perceive maternal health care provision in this region, the ways used to express needs in regard with maternal health care as well as your expectations for maternal health services. To do so ,we are going to ask you questions in regard with the topic. If there are questions or issues for which you do not wish to share your views because of any personal reason feel free to skip it. Anyway, you can stop this interview at any time you feel you cannot continue.  If you accept this interview, please express your views freely and try to describe the situation as it presents. In fact, your statements will be kept fully confidential and will never be reported to someone else. In this study, each participant will be identified by the mean of a code and the names will never appear. In addition, the information given will only be used by researchers and no one else.  To be able to gather properly your views and thoughts on the topic ,we are using a voice recorder. We cannot however record without your consent. The recorder will only be used in case you allow us to do so. Anyway ,the records will be erased and transcripts and notes will be kept by the researchers confidentially using codes.  The interview will take one hour. Only volunteers can participate in this study as nothing will be given as a reward to have participated in the study.  Accepting the interview is meant as one way among others to contribute towards improving voice for maternal health care provision in Burundi.  Could we start the recorder and commence the interview?  **Introduction**  Despite efforts made by the government to improve maternal health services performance and their utilization, figures yield from the Demographic and health survey in 2010 show that maternal health is still poorly established in Burundi:500 deaths per 100.000 live births which means that 5 women per 100.000 thousands live birth die while giving birth. Another figure we have is about health service utilization where we notice that 40% of women are still delivering at home.To address this situation, we are caring out a study to explore ways to strengthen community voice and especially women voice in order to use social accountability mechanisms to improve the performance of maternal health services. We think that this strategy can improve the performance of maternal health services and thus their utilization.  Could you tell us about yourself?  -What is your profession  -What is your age?  -Since when are you member or a responsible within this community  **Experience with organization of maternal health services**  -According to you what is needed to address health challenges especially those relating to maternal health care in Burundi?  - What kind of changes did you notice during last year regarding maternal health and family planning?  -What are the projects or programmes relating to maternal health are you caring out or implementing within your organization?  **If no such programme or project**   - What kind of message would you convey to your followers regarding maternal health care? - What is the message do you convey regarding Family Planning? - What kind of challenges do you face in providing maternal health services within faith-based facilities or when dealing with family planning issues?   **Experience with voice and social accountability**  -What kind of activities relating to health care do you usually organize at community level?  -What kind of ways do you usually use to get the feedback from the community regarding the quality of maternal health services you provide to the general community and to members of your religious community.  -How do you collaborate with health committee members?  -What kind of relationship do you have with community health workers?  -How do you usually proceed to denounce and point out weaknesses of maternal health services when you get opportunity to make an advocacy for their improvement ?  **Social and cultural factors influence on voice**  -As a religious leader do you think that general and maternal health care provided especially family planning are in keeping with the religious believes? Could explain Please.  -In our society in Burundi, do you think that it is easy for a woman to express a disagreement or dissatisfaction regarding the services received? Could you explain please?  -How can you help them?  -What are the factors in our culture prohibiting our population to express views especially those in regard with maternal health? |
